# Medical News

**Published:** 1851-09

**Authors:** 


### MEDICAL NEWS.

Meeting "of the Medical Association of Western Pennsylvania and Eastern Ohio."—Pursuant to previous notice, the above named organization held its second meeting in the Masonic Hall, at Sharon, Mercer Co., Pa. The Association having been called to order by the President, Dr. John Baskin, of Mercer, the minutes of the preliminary Convention held in Mercer, Pa., in May last, were read and approved.

The committee to whom had been entrusted the preparation of a Constitution and By-Laws for the Association, reported.

The report was received, and, its several sections having, on motion, been separately considered and voted upon, it was finally adopted and ordered to be transcribed into a book proper for the purpose, and the names of the members of the Association to be subscribed thereto. The preamble and the 1st and part of the 2d article of the constitution as adopted, specifying the objects, the title and the qualifications requisite for membership in the Association, are as follows, viz: "WHEREAS, We believe that the interests and prosperity of the medical profession, the progress and elevation of medical knowledge and science, and the welfare, health, and interests of the whole community, require a livelier spirit, a better acquaintance, more extended professional intercourse, and greater and more concerted action on the part of physicians, we do, therefore, now organize ourselves into a society that shall have for its great object the accomplishment of such ends, and adopt for our government the following constitution and by-laws, viz:

ART. 1st. SEC. 1st. This association shall be known and designated by the name and title of "The Medical Association of Western Pennsylvania and Eastern Ohio."

SEC. 2d. Any regular physician who has pursued a course of medical studies under the tutorship of a physician in good standing, and has attended one or more courses of lectures in a Medical School in good repute, and who, moreover, at the time of his application for membership, shall be actively engaged in the practice of his profession, shall be eligible to membership; (*provided,* that if he has not received a medical diploma from the school he may have attended, he shall, preparatory to being admitted, prepare a thesis on some medical subject, and pass favorably and creditably an examination on the same before the censors or a committee appointed by the President for that purpose,) and all physicians who have received the honorary degree of M. D. from a medical school in good standing, or who may have been in active practice for the last fifteen years, and who recognize and are governed by the principles and standard authorities of the regular profession, and conform to its code of ethics, as put forth by the American Medical Association, shall be eligible, and none others.

SEC. 3d. No irregular practitioner, under whatever name he may be classed, and no regular physician who uses or recommends any quack medicine or patent remedy, or who condescends to resort to finesse or trickery to gain practice, or who pretends to the possession and use of any extraordinary and secret remedies, or who will consult with, or in any manner countenance or encourage any irregular practitioner, shall be eligible to membership, and any member who shall be guilty of such practices, shall be reprimanded or expelled from the association.

On motion, the Association then entered into an election for officers for the ensuing year, which resulted in the choice of Dr. John Baskin, of Mercer, for President. Dr. John Mitcheltree of Mercer county, and

Dr. D. Leasure of Lawrence county, Vice Presidents. Dr. G. W. Baskin of Mercer, Recording Secretary. Dr. Wm. Wood of Lawrence Co., Corresponding Secretary. Drs. J. E. Stewart, R. M. Bebee, of Trumbull county, Ohio, John T. Ray, of Mercer Co., Pa., Walter W. Prentiss, of Mahoning Co., Ohio, Wm. R. Cowden, Butler Co., Pa., and W. G. Henderson, of Mercer Co., Pa., Censors.

Upon taking his seat, the President delivered an eloquent inaugural address, in which he dwelt upon the duties of, and advantages accruing to members of the Association.

Dr. Leasure of Lawrence county, previously appointed for that purpose, delivered an address to the Association, for which the thanks of the Association were tendered, and the address ordered to be published.

Dr. Hart of Clarksville, Pa., from the committee on epidemic diseases of the district, submitted an interesting report.

Dr. Henderson, of Middlesex, Pa., from the committee on obstetrics, submitted a lengthy and interesting report, the reading of which induced

Dr. G. W. Baskin to detail two cases of preternatural labor.

Dr. J. S. Stewart, of Brookfield, gave the history of a case in which a blighted fœtus was expelled at full term, together with a full grown child. Dr. Hart a similar case.

Dr. Irwin, of Sharon, communicated his favorable experience on the use of ext. belladonnæ in rigid os uteri, and it was confirmed by

Dr. Hart, who also advocated the use of chloroform under similar circumstances.

Dr. G. W. Baskin submitted a case of puerperal mania, in which he had successfully used the inhalation of ether to subdue the violence and overcome the restlessness of the patient.

Dr. W. R. Cowden, of Portersville, Butler Co., Pa., offered some interesting remarks on the use of nit. argent. in the cure of paronychia.

Dr. Leasure suggested the use of congelation in the treatment of hæmorrhoids, stating that in his hands it had been perfectly successful.

The following committees were then appointed by the chair, to report at the next meeting of the Association, viz:

On the use of cod liver oil, Drs. Cowden and Leasure. On anæsthetics, Drs. Hart and Rankin. On hæmorrhoids, Drs. Harnet and Dawson. On dysentery, Drs. Ray and Henderson. On eruptive diseases, Drs. Stewart and Bebee. On typhoid fever, Drs. Prentiss and Fowler.

By a provision in the constitution adopted, it also becomes the duty of every member of the Association to send in, or read to the Association, at every meeting, the details of two cases occurring in his own practice.

Drs. G. W. Baskin and Leasure, were appointed a committee of publication.

A committee consisting of one from each county represented in the Association, was appointed to give notice through the papers of the district, (each member in the papers of his own particular county,) of the time and place of the next meeting of the Association. The committee are,

| | | |
|---|---|---|
| Dr. John McCook, | of Columbiana Co., | Ohio. |
| " L. D. Kellogg, | Ashtabula " | " |
| " W. M. Prentiss, | Mahoning " | " |

| | | | |
|---|---|---|---|
| Dr. J. J. Wallace | Lawrence Co., | Pennsylvania, | |
| " W. R. Cowden, | Butler | " | " |
| " A. Sergeant, | Crawford, | " | " |
| " J. W. Riddle, | Venango | " | " |

Paid to the treasurer, by the members of the Association, thirteen dollars.

On motion, the Association then adjourned to meet at Youngstown, Mahoning County, Ohio, on the third Tuesday (the 21st day) of October next, at nine o'clock, A. M., punctually.

G. W. BASKIN, *Rec. Sec'y.*

AMERICAN MEDICAL ASSOCIATION. PRIZE ESSAYS.—At the meeting of the American Medical Association held in Charleston, S. C., in May last, the undersigned were appointed a Committee to receive and examine such voluntary communications, on subjects connected with medical science, as individuals might see fit to make, and to award a prize to any number of them, not exceeding five, if they should be regarded as entitled to such a distinction.

To carry into effect the intentions of the Association, notice is hereby given, that all such communications must be sent, post paid, on or before the first day of April, 1852, to Geo. Hayward, M. D., Boston, Mass. Each communication must be accompanied by a sealed packet, containing the name of the author—which will not be opened unless the accompanying communication be deemed worthy of a prize. The authors of the unsuccessful papers may receive them on application to the Committee, at any time after the first of June, 1852; and the successful ones, it is understood, will be printed in the Transactions of the Association.

GEO. HAYWARD, *Boston.*
J. B. S. JACKSON, "
D. H. STORER, "
JACOB BIGELOW, "
USHER PARSONS, *Providence, R. I.*

*Boston, Aug.* 20, 1851.

NEW HAMPSHIRE MEDICAL SOCIETY.—The following resolution was presented by Dr. J. S. Fernald, of Barrington, and unanimously adopted, viz:—

"*Resolved,* That it is inconsistent with the spirit of our code of ethics for Fellows of this Society to give certificates of approbation of medical compounds, whether the formulæ be known or not; and that such practice is detrimental to the interests of the faculty, and tends to foster empiricism."

Professors Crosby, Peaslee, and other members present, confessed their faults in this regard, and promised to sin no more.—*N. Y. Med. Gaz.*

NASHVILLE JOURNAL OF MEDICINE AND SURGERY.—We learn with pleasure that this well-stored and spirited Journal will be issued in *monthly* form, from the commencement of the second volume. Dr. BOWLING, the editor, informs us that his effort to establish a medical periodical in the State of Tennessee has been most handsomely responded to by the profession of Tennessee, and the West generally. We wish him continued success.

DR. VALENTINE MOTT, Jr., of N. Y., has been appointed to the Professorship of Surgery in the Washington University of Baltimore.

DR. PAUL F. EVE, of Augusta, Ga., has accepted the chair of Surgical Anatomy and Clinical Surgery in the Medical Department of the University of Nashville, Tennessee.

THE CHAIR OF MEDICINE IN THE FACULTY OF PARIS.—The "Concours" for this chair has lately been concluded by the appointment of Dr. Requin. The candidates were repeatedly balloted for, as they had all given proofs of great capabilities.

HONORARY DISTINCTIONS OFFERED TO M. RICORD.—This indefatigable laborer in his special branch of surgery, has lately received from the Sultan, the order of the Nizam, the cross of which, set with diamonds, is extremely valuable. The king of Spain has likewise wished to acknowledge the merit of M. Ricord, and has sent him the Order of Charles III.

THE STAFF OF THE HOTEL DIEU.—M. Husson, senior Physician to the Hôtel Dieu, has retired from practice, and his retreat has given rise to numerous changes in the hospital staff. Thus, M. Piedagnal goes to the Hôtel Dieu; M. Nonot to La Pitié; M. Barth to St. Antoine. There is likewise vacant the office of Physician to the Enfans Malades, and to the Female Venereal Hospital, besides the Presidency of the Faculty of Medicine, vacant by the resignation of M. Bérard, who has been quarrelling with his confrères.

DR. WORTHINGTON HOOKER has just received the *Fiske Fund Prize* for an Essay on Homœopathy, which is about being published. This is the second prize which Dr. Hooker has received from the same source, the first one having been awarded for a dissertation entitled the "History of Medical Delusions."

### DEATHS OF DISTINGUISHED PHYSICIANS.

Henry Jenner, M. D., nephew of the celebrated Jenner, died lately at Stone, near Berkeley, Gloucestershire, England, aged 83 years.

Dr. Moir, of Musselburgh, Scotland, died lately at Dumfries in that kingdom, in his fifty-fourth year. He was a skilful and distinguished practitioner, and a contributor of numerous popular poetical pieces to Blackwood's Magazine, under the signature of *Delta.*

M. Daguerre, inventor of the Daguerreotype, died near Paris, on the 10th of July.

Dr. Gilbert Smith, of New York, for more than fifty years a highly esteemed and popular practitioner of that city, died in West Chester Co., N. Y., on the 16th of July, aged 80 years.

The venerable Sir Geo. Smith Gibbes, M. D., died on the 23d of June, at Sidmouth, Eng., aged 80. He was a Fellow of the Royal College of Physicians, and for many years physician extraordinary to Queen Charlotte, during whose reign he was knighted.

M. Amussat on the Employment of Water in Surgery.—This valuable essay, translated by Professor Hamilton, of the University of Buffalo, and originally published in the Buffalo Medical Journal, has, we are glad to see, been issued as a distinct publication.

Trial for Alleged Malpractice.—Prof. T. Spencer, of Milwaukee, was lately tried on a charge of malpractice on a little girl between four and five years of age; the declaration being substantially, that "large doses of calomel," "negligence," "want of skill," &c., had produced necrosis or mortification of the lower jaw. The verdict of the jury in the case was—"No cause of action."

"After the trial, the immediate neighbors and professional friends of Prof. Spencer, gave expression of their feelings of gratification, at a large meeting assembled for the purpose, when the following Preamble and Resolutions were unanimously adopted:

"Inasmuch as we approve of the justness of that law which holds members of our profession accountable for the correctness of their practice, as due not only to the patient who may have been aggrieved, and to the protection of the community at large, but always as the strongest test which society can apply to distinguish between science and quackery, therefore,

"*Resolved,* That as our Legislators have denied us the right to apply a test between science and empiricism, in season to prevent the injuries of the latter in the community, we hail with pleasure the application, in all proper cases, of the remedy afforded in *common law*, for redress of such injuries, *after they are committed;* but at the same time, cannot too highly censure the conduct of any, who, by false and improper representations, encourage an appeal to this law for redress of fancied injuries.

"*Resolved*, That, with unmingled pleasure, we tender to Professor Spencer, our congratulations on the result of the trial through which he has just passed.

"*Resolved*, That while we congratulate Professor Spencer on his unanimous acquittal from blame, we also deeply sympathize with the family of Mr. Russel, in the grievous and distressing affliction to which they have been subjected, and assure them of our sincere sympathy and condolence."

Enormous Corpulence.—Dr. Aran lately published in *L'Union Médicale*, the case of a woman of twenty-five, under the care of M. Rostan, at the Hôtel Dieu, in Paris, who died one month after admission, evidently from a surprising accumulation of fat. Bronchitis and dyspnœa were prominent symptoms. She could not have weighed less than 400 pounds, every portion of her body being disfigured by deposition of adipose matter. On a post-mortem examination, fat was found both in the chest and abdomen; even in the mediastina and between the pleura and the walls of the chest. The viscera of the abdomen were literally lost in the fat, but no organ had undergone fatty degeneration. The patient could not account for the polysarcia with which she was affected, and had lived very moderately.—*London Lancet.*

The Sweating Sickness.—The sweating sickness continues to prevail with great intensity in the South of France. In a few hours fatal congestion of the brain often ensues. Quinine in large doses has been found to be the best remedy.

The Hunterian Museum.—In the parliamentary grants of money to medical institutions and charities, voted by the House of Commons, on the 19th July, will be found one of £15,000 towards the erection of an additional museum, and for enlarging the theatre of the Royal College of Surgeons, for the delivery of the Hunterian Lectures. The vote for this munificent sum was agreed to by the House without a division, the Chancellor of the Exchequer stating, that many years ago Mr. John Hunter, the most celebrated surgeon, perhaps, which this country ever produced, accumulated a collection of anatomical specimens of great value, which eventually became so extensive and valuable, that they were bought by the country, and ultimately committed to the care of the College of Surgeons. Since that museum had been committed to their care, the College of Surgeons has most faithfully discharged the trust reposed in them. They had themselves greatly added to the museum, at a cost of no less than £189,000, and thrown it open to the public. Having spent that very large sum of money, which they were not bound to do, they found themselves without adequate funds to enlarge the museum and theatre, as was their wish. Under these circumstances, they had applied to Government for assistance. The following sums were awarded to medical charities in Ireland :—To the Fever Hospital, Cork-street, Dublin, £3,040 ; to the Westmoreland Lock Hospital, Dublin, £1750; to Dr. Stevens's Hospital, £1200; the Dublin Lying-in-Hospital, £600; and to the Hospital for Incurables, Dublin, £400,—making a total of £6990 to the medical charities of Dublin. At the same time the sum of £9969 was voted to defray the charges of the General Board of Health, not without great opposition.—*London Lancet.*

Testimonial to Dr. Laycock of York.—A handsome silver tea service has been presented to Dr. Thomas Laycock of York, England, by the Associated Licentiates Extra Urbem of the College of Physicians, with the following inscription :

"Presented to Thomas Laycock, M. D., Physician to the York Dispensary, Lecturer on the Practice of Medicine in the York Medical School, &c. &c., by his friends, the Associated Licentiates Extra Urbem of the Royal College of Physicians of London, in testimony of their very cordial esteem and regard, and in gratitude for the ability, energy and zeal with which he has maintained their rights and interests.—Anno Domini, 1851."—*Dub. Med. Press.*

Poisoning by Nicotine.—An extraordinary trial, which took place in Belgium, and in which the Count and Countess Bocarmé were accused of poisoning the Countess' brother, by the forcible administration of nicotine, has been recently brought to a conclusion. The Count, who was found guilty, was condemned to death, and has since been executed; the lady may consider herself fortunate in having escaped. The most interesting circumstances connected with this horrible affair are, that the Count seems to have prepared the poison with his own hands; and that although he probably selected nicotine as a substance which could not be chemically detected in the body of his victim, M. Stas, a Belgian chemist, obtained distinct evidence of its presence on applying suitable tests. M. Orfila has since communicated the results of some experiments on the detection of nicotine, to the French Academie de Médecine, and although the process which he recommends differs slightly from that which was adopted by M. Stas, he arrives at the same conclusion, viz., that the poison can be detected unequivocally if present even in the quantity of a few drops in the stomach and bowels, and that it may even be found in the liver and other organs after its absorption into the system.—*London Monthly Journal.*

Bell's Grave.—Perhaps it may not be uninteresting to the admirers of the great Sir Charles to hear of his resting place. On the western bank of the Severn, and about four miles north of Worcester, we come to a rising ground, on which stands a large yellow building, surrounded with trees, and called Kallow Park. In this house Sir Charles died quite suddenly, and on the evening of the day of his arrival; it is supposed that a rupture of some of the heart's cavities caused this great loss to the medical world, but Lady Bell would not allow an autopsy, and thus we are undecided as to the immediate cause of his death.

As we approach the park and ascend the pathway, we see the village church, and in the south-west corner of the yard a few plain tombs are visible, surrounded with iron rails; on one of them the following lines are inscribed:

Sir Charles Bell, K. T.
Born at Edinburgh, MDCCLXXV.
Died at Kallow Park, April xxix., MDCCCXLII.
"Blessed are the pure in heart for they shall see God."
This stone is placed by Martha, his widow.

Within the church, and almost opposite the entrance, and on the north side of the communion table, is a white marble stone, plain and unadorned, and written on its surface in black letters these words:

"Sacred to the memory of Sir Charles Bell, who, after unfolding with unrivalled sagacity, patience and success, the most wonderful structures of our mortal bodies, esteemed lightly his greatest discoveries except only as they tended to impress himself and others with a deeper sense of the infinite wisdom and ineffable goodness of the Almighty Creator. He died while on a visit of Friendship at Kallow Park."

*Dublin Med. Press.*

